# Does Papillary Carcinoma of Thyroglossal Duct Cyst Develop De Novo?

**DOI:** 10.1155/2015/382760

**Published:** 2015-08-16

**Authors:** Tekin Baglam, Adem Binnetoglu, Ali Cemal Yumusakhuylu, Berat Demir, Gokce Askan, Murat Sari

**Affiliations:** ^1^Department of Otorhinolaryngology-Head and Neck Surgery, Pendik Training and Research Hospital, Marmara University Medical Faculty, Mimar Sinan Caddesi No. 41, Fevzi Cakmak Mahallesi, Ust Kaynarca-Pendik, 34899 Istanbul, Turkey; ^2^Department of Pathology, Pendik Training and Research Hospital, Marmara University Medical Faculty, Mimar Sinan Caddesi No. 41, Fevzi Cakmak Mahallesi, Ust Kaynarca-Pendik, 34899 Istanbul, Turkey

## Abstract

*Backround*. Thyroglossal duct cyst (TDC) is a developmental abnormality of the thyroid gland. Due to embryological remnants of thyroid tissue located in the TDC, the same malignant tumors that develop in the thyroid gland can also develop in the TDC. *Methods*. We present the unique case of a 39-year-old female with simultaneous de novo papillary carcinoma in a TDC and the thyroid gland. *Results*. With the suspicion of simultaneous papillary carcinoma in the TDC and the thyroid gland, Sistrunk procedure with total thyroidectomy and central neck exploration was performed. *Conclusion*. The clinician should have a high index of suspicion upon encountering papillary carcinoma of the TDC to differentiate de novo papillary carcinoma in the TDC from those originating from the thyroid gland, because papillary carcinoma in TDC may originate from an occult thyroid papillary carcinoma.

## 1. Introduction

Thyroglossal duct cyst (TDC) is a developmental abnormality of the thyroid gland during the embryologic period. The thyroid gland originates from the base of the tongue and migrates to its normal anatomic localization in the neck through the thyroglossal tract. After the migration phase, the thyroid gland completes its maturation and the thyroglossal tract atrophies. If the canal does not atrophy completely, a TDC evolves. TDC is the most common congenital mass found in the neck midline; however, 10% of TDC can also be found in the lateral neck [1–4]. The TDC can be found along the neck midline in the thyrohyoid (60%), suprahyoid (2%), suprasternal (13%) and intralingual (25%) areas [2, 3, 5]. Due to embryological remnants of thyroid tissue located in the TDC, the same malignant tumors that develop in the thyroid gland can also develop in the TDC. Papillary type thyroid carcinoma can develop in the TDC de novo with an incidence of 1%, although some authors believe that the thyroglossal duct instead serves as a natural route for occult thyroid carcinoma metastases [6–8]. Thyroid carcinomas originating from the TDC are mostly seen in females between the ages of 20–60 years and present as a complaint of pain at the level of the thyrohyoid membrane along the neck midline with a physical examination finding of a palpable mass without tenderness [9]. Occasionally, the presenting sign of thyroid carcinoma is a rapidly growing mass, mimicking TDC infections. In this case, diagnosis is usually made after histopathologic examination of a specimen from a Sistrunk resection [10]. Ultrasound (US), computed tomography (CT), magnetic resonance imaging (MRI), and fine-needle aspiration biopsy (FNAB) can be useful techniques to differentiate TDC carcinomas from simple TDC before a surgical operation. Typically psammoma bodies found in FNAB and irregularly bordered solid nodules found on radiologic screens are identified as the best targets for a diagnostic resection of thyroid carcinoma. Papillary carcinomas originating from the TDC rarely metastasize to distant sites; thus the prognosis is similar to that of papillary carcinoma of the thyroid gland. Herein, we report a case of primary papillary thyroid carcinoma originating from the TDC accompanied by occult thyroid papillary carcinoma originating from the thyroid gland simultaneously.

## 2. Case

**Figure 1:**
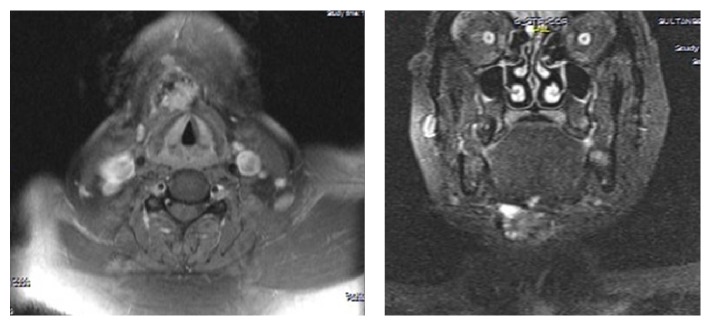
Axial and coronal section MRI demonstrating a solid soft tissue mass containing high signal hyperintensity, cystic changes, and invasion of the infrahyoid muscles.

A 39-year-old female was admitted into our clinic with a complaint of a 3-month history of a slowly growing mass along the neck midline. The physical examination revealed a 3 × 1.5 cm immobile mass along the neck midline over the thyrohyoid membrane, accompanied by a 3 × 2 cm palpable nodule in the left thyroid gland. A neck US revealed a calcified heterogenic-hypoechoic solid lesion of 30 × 18 mm at the level of the hyoid bone in the submental area, which was not related to the thyroid gland. A thyroid gland US revealed several hypoechoic nodules, the largest being 38 × 24 mm, with some having micro calcifications in the left lobe of thyroid gland, while the study identified no right lobe lesions. FNAB was performed on both the midline neck mass and the left lobe nodule. The FNAB results were reported as being consistent with papillary thyroidal carcinoma for the mass along the neck midline and as benign cytology for the nodule in the left thyroid lobe. MRI of the neck showed a 30 × 20 mm midline solid soft tissue mass with hyperintense signal after injection with contrast media. The MRI also demonstrated cystic changes in the mass and evidence of invasion into the infrahyoid muscle group (Figure 1). After considering all of the clinical and radiological data, despite a histopathologic diagnosis of benign cytology for the left lobe nodule, we performed a Sistrunk procedure with total thyroidectomy and central neck exploration due to suspicion of simultaneous de novo development of papillary carcinoma in the TDC and the thyroid gland. Informed consent was obtained prior to the procedure.

**Figure 2:**
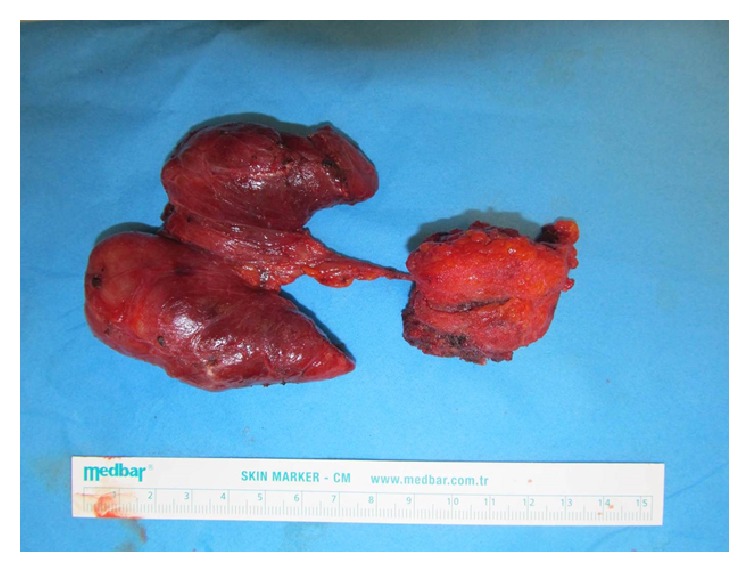
Specimen after total resection of the TDC and thyroidectomy with suspected papillary thyroid carcinoma.

During the Sistrunk procedure, a solid mass invading the infrahyoid muscle group was encountered, and gross total resection of the tumor was performed. A total thyroidectomy operation was also conducted (Figure 2). During central neck exploration, samples of the suspected lymph nodes were sent for frozen section pathology, the results of which confirmed the diagnosis of papillary carcinoma metastasis. After completion of the exploration, the central neck was dissected and additional specimens were sent to pathology. Pathologic examination identified the thyroid specimen as containing papillary carcinoma of the diffuse sclerosing type and papillary carcinoma of the classical type for the TDC specimen.

**Figure 3:**
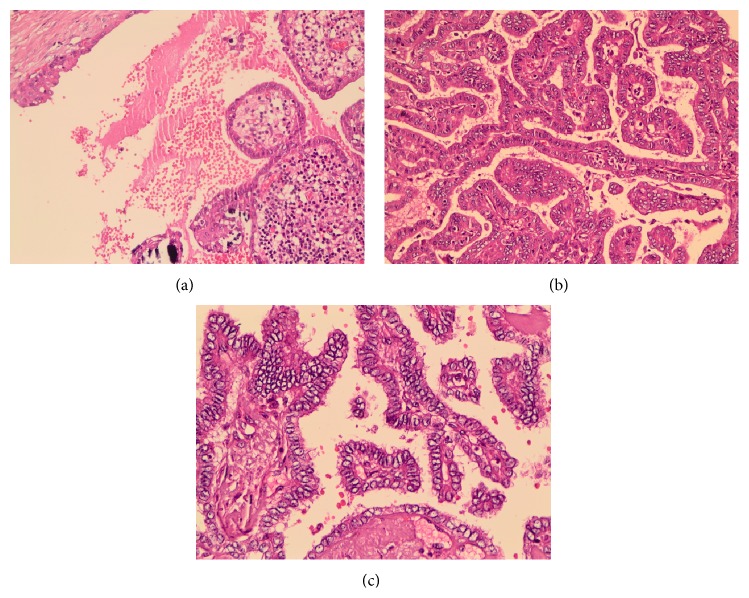
(a) The tumor is within a cystic structure which is lined by stratified squamous epithelium (×200, H&E). (b) Papillary structures which are typically complex branching, covered by epithelium with disturbed polarity and eosinophilic cytoplasm (×200, H&E). (c) Nuclei are large, oval shaped and show clearing and some have grooves (×400, H&E).

**Figure 4:**
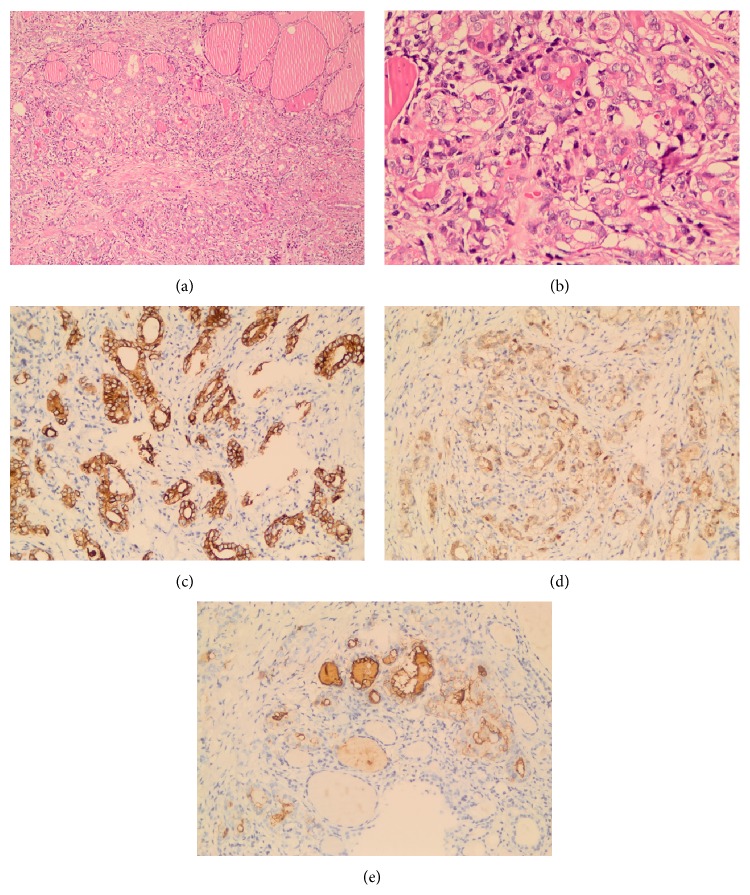
(a) Tumor has papillary architecture which is <1 cm with desmoplastic stroma and normal thyroid follicles identified, the upper right side (×200, H&E). (b) Nuclei are large and oval in shape and show clearing (×400, H&E). (c) The tumor cell cytoplasm is strongly reactive with CK19 (×200, CK19). (d) Galectin-3 staining is confined to the cytoplasm of the tumor cells (×200, Galectin-3). (e) HBME-1 staining is present within the plasma membrane of the tumor cells (×200, HBME-1).

Histopathology revealed the nodule which was seen on the cut section of thyroglossal cyst tissue; the tumor was located within cysts, composed of complex branching true papillae that contain fibrovascular stalk (Figures 3(a) and 3(b)). The papillae were lined by neoplastic epithelial cells with enlarged, clear “Orphan Annie eye” nuclei and some of them had nuclear grooves (Figure 3(c)) that were characteristics for papillary thyroid carcinoma, classical variant. The tumor was unencapsulated with 3,5 cm in diameter. The histopathological examination of the nodule that was seen on the cut section of left lobe of thyroid gland was as follows: unencapsulated, with a 2 mm diameter of lesion being determined (Figure 4(a)). The tumor cells were composed of large, oval shaped and clear nuclei with some having a groove which was also characteristic for papillary carcinoma (Figure 4(b)). Because of the tumor size that was smaller than 1 cm in diameter and the presence of dense desmoplastic stroma, we described it as diffuse sclerosing variant of papillary microcarcinoma. Immunohistochemistry results showed that the tumor cells were positive for CK 19, Galectin-3, and HBME-1 which was compatible with papillary carcinoma (Figures 4(c), 4(d), and 4(e)).

After the operation, a radioactive iodine (RAI-131) dose of 150 mCi was administered as adjuvant therapy. After RAI treatment, the patient had an undetectable thyroglobulin level at the 6-month follow-up. In addition, an I-131 whole body scan dose of 5 mCi revealed no pathologic involvement. Both a thyroglobulin level and an I-131 whole body scan were planned during follow-up at 1, 2, and 5 years after the initial RAI adjunctive therapy.

## 3. Discussion

TDC is the most common congenital mass along the neck midline. Papillary carcinoma is the most common malignant tumor of both the TDC and the thyroid gland [11]. Papillary carcinoma originating from the TDC usually manifests a slowly growing mass along the neck midline with a low propensity for distant metastases and having good prognosis. Papillary carcinoma from the thyroid gland also has good prognosis. A controversial issue is whether papillary carcinoma in the TDC develops de novo or as a type of metastasis from an occult tumor nidus in the thyroid gland [9, 10, 12–14]. A limited number of reported cases describe the presentation of a TDC papillary carcinoma accompanied by a thyroid gland papillary carcinoma [6–8, 15–17]. It may be difficult to differentiate primary papillary carcinoma of the TDC from metastasis of an occult carcinoma of the thyroid gland. This differentiation, however, can play a crucial role in the treatment strategy in terms of including thyroidectomy as part of the treatment strategy versus TDC total resection alone.

In our case, the papillary carcinoma in the TDC was classical in type whereas that in the thyroid gland was diffuse sclerosing type, arguing against metastasis through the thyroglossal duct as a natural route of spread for an occult thyroid carcinoma. Rather the findings suggest primary papillary carcinoma of the TDC with a “de novo” origin. The preoperative evaluation—including detailed head and neck examination, thyroid function tests, and radiologic and FNAB—is important for differentiation of these clinical cases. The incidence of thyroid carcinoma in the TDC is low, ~1%. However, the frequency of thyroid carcinoma in the TDC seems to be increasing; indeed, several cases in which nodules were also found within the thyroid gland have been reported, making thyroid gland evaluation an important component of the workup [14]. The success of a definitive surgical procedure for papillary carcinoma of the TDC is dependent on the preoperative radiologic and histopathologic evaluation, as well as the intraoperative tumor evaluation [16, 17]. A Sistrunk procedure with clear surgical margins and adjunct treatment with RAI is the standard regimen for TDC papillary carcinoma [9]. Total thyroidectomy and central neck dissection should be added to the treatment when a nodule is found in the thyroid gland during inspection, palpation, and/or intraoperative evaluation [9]. Surgery, external radiation therapy, or radioactive iodine are the treatments of choice in patients with local relapse or metastasis.

## 4. Conclusion

It is rare to encounter papillary carcinoma in both the TDC and the thyroid gland concurrently. The clinician should have a high index of suspicion upon encountering papillary carcinoma of the TDC to differentiate de novo papillary carcinoma in the TDC from those originating from the thyroid gland, because papillary carcinoma in TDC may originate from an occult thyroid papillary carcinoma, which must be treated by thyroidectomy.
